# *Chlamydia trachomatis* invasion: a duet of effectors

**DOI:** 10.1042/BST20240800

**Published:** 2025-03-24

**Authors:** Tyler J. Zimmerman, Rey A. Carabeo

**Affiliations:** Department of Pathology, Microbiology, and Immunology, University of Nebraska Medical Center, Omaha, Nebraska 68198, U.S.A.

**Keywords:** Chlamydia trachomatis, Invasion, Type III Effector, Actin Dynamics

## Abstract

Members of the genus *Chlamydia* require an intracellular niche for growth and replication, thus highlighting the extreme significance of its ability to invade epithelial cells—the favored host cell *in vivo*. Because epithelial cells are not phagocytic, the uptake of *Chlamydia* must be driven by the pathogen. To this end, two bacterial proteins, translocated actin-recruiting protein (TarP) and translocated membrane effector A (TmeA), identified in *Chlamydia trachomatis* are translocated from the infectious chlamydial elementary bodies to the host cell cytosol to facilitate extensive remodeling of the cortical actin network to produce protrusive structures designed for pathogen engulfment. Notably, both effectors act by promoting highly localized actin nucleation at sites of bacterial adhesion. However, they have non-redundant functions, with both required for optimal actin remodeling dynamics and efficient invasion. Finally, these effectors also mediate the latter stages of the invasion process, specifically by modulating host dynamin 2, a large GTPase critical to closure and scission of invaginating vesicles harboring elementary bodies. In summary, TarP and TmeA orchestrate major aspects of *C. trachomatis* invasion.

## Background

*Chlamydiae* are obligate intracellular bacteria with high clinical relevance [1]. It is estimated that over 100 million new cases of the sexually transmitted disease chlamydia are reported globally, which represents a significant public health burden [2]. In addition, *Chlamydia trachomatis* is the causative agent of the most prevalent preventable blindness, trachoma, which disproportionately affects women and children in underdeveloped countries [3].

From a microbiology perspective, the intracellular niche established in epithelial cells enables complex interactions of *Chlamydia* with its host cell [4]. Foremost is metabolic interaction, which compensates for the relatively small 1 million basepair genome of the bacterium, that through evolution dispensed of several metabolic genes [5], leading to dependency of the bacterium on its host for nutritional requirements. The presence of essential metabolites is not sufficient, as they also need to be delivered to the bacterium; and in this regard, *C. trachomatis* utilizes a collection of proteins that regulate the interaction of its vesicular niche called an inclusion with host organelles and nutrient-laden vesicles [5].

Chlamydial dependence on its host represents a point of vulnerability that often leads to either aberrant growth of the viable, but non-replicating bacterium, or death and elimination by the host cell through degradation. Host-mediated antibacterial mechanisms encountered by *Chlamydia* include nutritional immunity [6], which is the process of restricting access to essential nutrients of pathogens; and mechanistically, this is achieved via multiple means that include nutrient catabolism and reduced expression of host components needed to acquire and transport metabolites. Both processes are often part of the host’s overall downstream response to specific cytokine signals [7,8]. Despite several mechanistically distinct anti-chlamydial arsenal of the host, *C. trachomatis* remains a successful human pathogen, underscoring an effective mechanism to handle host strategies designed to limit pathogen growth, replication, and survival. However, *Chlamydia* can only do this from inside cells and within its protective and replicative inclusion niche. In other words, the inclusion, when fully modified by the pathogen through insertion of several inclusion membrane proteins (Incs), becomes the chlamydial command center where it manipulates various host cell processes for its benefit. The importance of the inclusion points to an absolutely crucial role of invasion to enable *Chlamydia* to gain access to the intracellular environment and establish its niche. Therefore, *Chlamydia* pathogenesis is underpinned by efficient invasion.

### A brief overview of *Chlamydia* adhesion

The adherence of *C. trachomatis* to the surface of epithelial cells is a two-step process [9]. The overall positive charge of *C. trachomatis* biovar lymphogranuloma venereum (LGV) serovar L2 facilitates the initial electrostatic interaction with negatively charged glycosaminoglycans on the host cell surface [10,11]. This step is reversible, but only very transiently because it is immediately followed by a series of molecular steps that initiate rapid internalization of the infectious particle called the elementary body (EB). However, the progression of the adhesion process to internalization can be halted by performing inoculation of cell monolayers *in vitro* at 4°C. In this infection condition, EBs can be dissociated from cells by soluble heparin [9], and internalization can be synchronized by the addition of media pre-warmed to 37°C. EB internalization is preceded by an irreversible binding step [9], which is dissociable only by trypsinization [12]. Importantly, stable irreversible binding requires the initial electrostatic interaction, presumably to stabilize the chlamydial EB on the cell surface to enable irreversible interaction [9]. This multistep process of adhesion is reminiscent of several viral and bacterial pathogens. For example, the interaction of herpes simplex virus (HSV) with its target host cell involves conformational changes to viral glycoproteins, which are preceded by binding to cell surface proteoglycans [13]. Thus, it appears that microbial pathogens have evolved to leverage a conserved feature of glycating proteins destined for display on the cell surface. Several chlamydial proteins displayed on the EB surface have been proposed to mediate its reversible interaction with host proteoglycans, and currently available data point to functionally redundant adhesion factors. Unlike in HSV where the viral factors mediating interactions with host cell proteoglycans are already known, the identity of the chlamydial factors remains unclear. This is mainly due to the sheer number of candidates proposed [14], in addition to the limited robustness of the assays for function that was at the time limited by the lack of an established molecular genetic system, including transformation for *C. trachomatis*.

Notably, irreversible adhesion involves a genetically encoded, unidentified host factor. A Chinese Hamster Ovary (CHO)-mutant cell line selected for resistance to *Chlamydia* infection supported electrostatic interaction but was unable to establish irreversible attachment [9] and thus was also inefficient in inducing actin remodeling, which is discussed in greater detail below, and internalization. In wildtype cells, stable adhesion results in the formation of actin-rich cell surface projections [9,15] that is mediated by chlamydial proteins or effectors translocated across the host cell plasma membrane via specialized needle-like apparatus termed the type III secretion system (T3SS). With the aid of translocation chaperones, type III effectors are passed through the apparatus and the needle that contacts the host plasma membrane and into the host cell cytosol where they act to subvert cellular processes [16]. They include the actin remodeling machinery. Modulation of actin recruitment, polymerization, and turnover combines to form hypertrophic microvilli that surround and eventually engulf adhered bacteria [15]. The internalization of *C. trachomatis* is rapid and efficient, with approximately 80% of infectious EBs internalized within 10 min [15].

### Manipulation of actin dynamics by invasion-associated type III effectors

The cortical actin network of epithelial cells undergo dynamic changes in response to growth factors or during receptor-mediated endocytosis [17,18]. Microvillar hypertrophy that was clearly observed in infected cells by scanning electron microscopy [15] is a hallmark of *C. trachomatis* invasion and is dependent on actin polymerization [15], with the cytochalasin D treatment leading to their disassembly, inhibition of formation, and EB internalization [15]. Cytochalasin D washout led to the preferred reformation of hypertrophic microvillia at the sites of EB adhesion, indicating localized signal that originates from the bacterium. Subsequent, more detailed investigation of this process led to the identification of pre-synthesized EB-associated effectors that are translocated to the host cytosol where they integrate within host cell signal transduction pathways linked to actin remodeling.

The first invasion-associated T3SS effector identified is the translocated actin-recruiting protein (TarP) of *C. trachomatis* LGV serovar L2 [19]. It is encoded within an approximately 3-Kbp open-reading frame that is located within the ‘plasticity zone’ of the *C. trachomatis* genome, where a significant portion of genetic polymorphisms within the genus *Chlamydia* are located [20]. There is high degree of variation in protein size (~700–1200 amino acids) [21]. For the homolog of *C. trachomatis* serovar L2/434/Bu, the best-studied strain, the 1005-amino acid polypeptide, exhibits interesting features, specifically the presence of domains resembling eukaryotic protein–protein interaction motifs. Starting from the N-terminal half, there is a repeated unit of approximately 50 amino acids, termed the phosphodomain (PD), which is phosphorylated at specific tyrosine residues [19,22,23]; a proline-rich domain (PRD) implicated in *in vitro* TarP oligomerization into a 13-mer complex [24]; a G-actin-binding motif (ABD) [25]; and an F-actin-binding domain (FABD) [26]. The ABDs overlap sites for interaction with the focal adhesion kinase (FAK) (Leu/Asp; LD domain) and vinculin (vinculin-binding domain; VBD) [24,27], leading to two different models for how TarP functions in actin remodeling – as a signaling scaffold and as a bacterial actin nucleator [22,24,27,28].

#### TarP as a signaling scaffold

**Figure 1:**
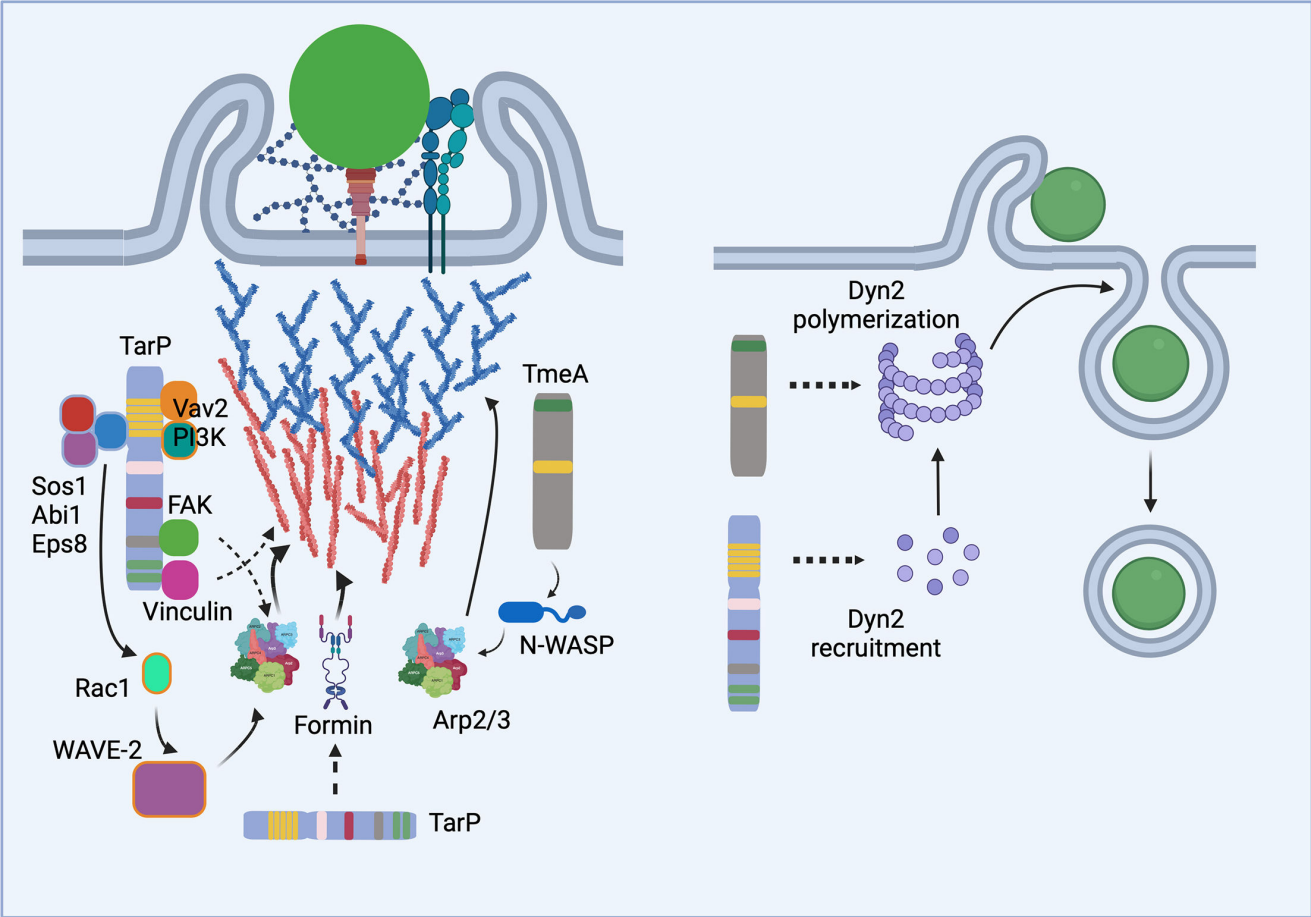
An updated model of the signaling events during *C. trachomatis* invasion. *Chlamydia* (green circles) bind to heparan sulfate proteoglycans and host cell surface receptors. The T3SS contacts the plasma membrane and translocates the effectors TarP and TmeA. TarP serves as a signaling scaffold for several host signaling proteins after phosphorylation by host tyrosine kinases (not shown). Note the multiple means of activating the Arp2/3 complex. Formin family members are also involved; while their recruitment is TarP-dependent, the exact mechanism is uncharacterized. Arp2/3 and formins co-operate to establish the TarP-associated actin network (orange). The other effector TmeA binds and activates N-WASP, which in turn activates Arp2/3. This signaling module promotes the formation of the TmeA-associated actin network (blue). Consistent with experimental model, this network is built upon and indeed, dependent on the TarP actin network. The diagram on the right illustrates the roles of TarP and TmeA in invasion events downstream of actin remodeling. TarP is responsible for recruiting dynamin-2 to sites of invasion, while TmeA indirectly promotes dynamin-2 functional activation, probably via polymerization to enable the formation of collars around the necks of invaginating *Chlamydia*-containing vesicles. TarP, translocated actin-recruiting protein; TmeA, translocated membrane effector A; T3SS, type III secretion system. Created in BioRender. Romero, M. (2024) https://BioRender.com/r69t869.

TarP-mediated signaling originates from both the N-terminal PD and the LD/VBD motifs in the C-terminal half of the protein [22,24,27] (Figure 1). The former is phosphorylated at specific tyrosine residues by members of the Src family, Abl, or Syk kinases [23,29,30]. Mouse embryonic fibroblasts ablated for Src, Yes, and Fyn (SYF-/-) yielded lower levels of phosphorylated TarP in Western blot assays [[[23]]]. Similarly, the inhibition of Abl activity in SYF-/- cells reduced but did not eliminate TarP phosphorylation completely [30]. A similar observation was made for Syk [30]. Results indicate that additional tyrosine kinases are involved. From co-immunoprecipitation studies using custom synthesized phosphopeptides (DAAAD**Y**EPISTTENI**Y**ESIDDSSTSDPENTSGGAAALNSLRGSSYSNYD) with only one of two tyrosine residues (bolded and underlined) per PD unit phosphorylated, Sos1 exhibited binding preference to the first phosphotyrosine residue, while Vav2 bound equally well to both [22], indicating functional distinction and non-redundancy of the tyrosine phosphorylation events.

The immediate outcome of TarP interaction with Sos1/Eps8/Abi1 or Vav2/PI3K is the activation of the Rac/WAVE2/Arp2/3 pathway of actin remodeling. The molecular complex that maintains WAVE2 inhibition is disassembled upon interaction with GTP-bound (i.e., active) Rac1, enabling the liberated WAVE2 protein to interact with monomeric G-actin and the Arp2/3 complex. WAVE2 bringing G-actin and Arp2/3 in close proximity facilitates actin nucleation and subsequent polymerization (Figure 1)

The activation of SYF, Abl, and Syk often requires upstream signaling steps [31] and, thus, could be essential to the functional scaffold role of phosphorylated TarP. *Chlamydia* invasion is associated phosphorylation of receptors. EphrinA2 (EphA2) has been implicated in *Chlamydia* binding to host cells [32], with receptor phosphorylation and activation of PI3K, raising the possibility that EB/EphA2 interaction is bona fide binding event by activating validated invasion-relevant signaling. However, its link to the activation of SYF, Abl, and or Syk has not been fully characterized. Therefore, association with invasion could only be implied by EphA2’s activity toward Src and Fyn kinases. The FGF2-FGFR pathway has also been implicated in triggering invasion-related signaling pathway. The mechanism proposed invokes a mechanism where FGF mediates the interaction of the EB with FGFR by acting as a bridge [33]. Because neither FGFR nor the EphrinA2 receptor has been directly implicated in the actin recruitment and remodeling aspect of *Chlamydia* invasion, evidence remains circumstantial. In short, details of how host receptor tyrosine kinases are functionally linked to TarP-mediated signaling to the actin remodeling machinery remain poorly characterized.

Illustrating protein–protein interactions of TarP that are independent of its phosphorylation is the binding of FAK and vinculin to the LD and VBD motifs, respectively [24,27]. These domains are conserved in *trachomatis* and non-*trachomatis* species, which potentially implicates them as an alternative means of remodeling actin. Indeed, the knockdown of either FAK or vinculin by RNA interference decreased invasion efficiency of non-*trachomatis* strains that possess TarP homologs lacking the PD. The TarP-FAK signaling axis depends on Arp2/3, but whether it does so independent of WAVE2 has not been addressed. Similarly, how TarP–vinculin interaction promotes actin polymerization and remodeling is unelucidated. In support of FAK and vinculin playing a role in invasion, a large unbiased RNA interference screen in the Drosophila S2 cell line for host genes required for efficient *Chlamydia muridarum* invasion identified the two proteins [29]. Further highlighting their significance to *Chlamydia* invasion, a recently identified chlamydial adhesin, named Ctad1, was characterized to interact with host β-integrin [34]. Whether this leads to signaling through FAK and/or vinculin was not addressed. In intact mucosal epithelia, β-integrin is localized on the basolateral surface, which brings into question accessibility to Chlamydiae located at the apical side lining the genital tract lumen. However, damage to the epithelium would expose β-integrin receptors to provide a specific scenario where Ctad1-β-integrin could be relevant. Whether the Ctad1/β-integrin signaling interacts with the TarP/FAK/vinculin axis remains to be investigated.

#### TarP as a bacterial actin nucleator

The actin-nucleating activity of TarP depends on the oligomerization domain and the ABDs [28]. *In vitro* pyrene actin assays demonstrated that TarP oligomerization compensates for the absence of multiple ABDs. Oligomerization positions multiple ABDs to theoretically increase the local concentration of monomeric actin and overcome the thermodynamic barrier associated with the formation of actin trimer nucleators. In support of this model, actin nucleation by TarP homologs with more than one ABDs bypassed PRD requirement.

It was proposed that signaling and actin nucleation may not be mutually exclusive and, instead, co-operate to promote robust actin recruitment [35]. Examination of products of *in vitro* TarP-mediated actin polymerization revealed a bias toward filamentous actin, which could potentially serve as binding sites for Arp2/3 and formation of branched actin network. Indeed, *in vitro* pyrene actin assay demonstrated synergism in actin polymerization between purified preparations of TarP and Arp2/3 [35]. Recent genetic studies revealed that the PRD and ABD motifs were dispensable [36] based on the genetic complementation of a Δ*tarP* strain. Suboptimal invasion was reversed by the infection of the TarP-deficient strain that has been complemented with a plasmid-encoded TarP derivative lacking either the PRD (TarPΔPRD) or ABD (TarPΔABD). In addition, an incomplete rescue of invasion efficiency was observed in a Δ*tarP* strain complemented with TarP lacking the phosphodomain (TarPΔPD), thus favoring the signaling scaffolding model for TarP.

Despite the dispensability of the PRD and ABD domains, filamentous actin polymerization was determined to be still relevant to invasion, but their polymerization depended on members of the formin family of nucleators, specifically Fmn1, DIAPH2, and DIAPH3 that are recruited to sites of invasion [37] (Figure 1). Their inhibition via treatment with the pan-formin inhibitor SMIFH2 decreased invasion efficiency and negatively affected actin dynamics. Furthermore, formin inhibition also negatively influenced the rate of Arp2/3 recruitment, consistent with filamentous actin acting as binding sites for Arp2/3 complexes. This collaboration was found to be bidirectional, with Fmn1 recruitment depending on Arp2/3 function. Pharmacological inhibition of Arp2/3 activity with CK-666 decreased recruitment of Fmn1 at invasion sites. In summary, the bulk of actin nucleation observed during invasion is likely the result of TarP functioning as a signaling scaffold to activate Arp2/3- and formin-nucleated actin polymerization.

#### TmeA is an effector that activates N-WASP

A fascinating finding further highlighted the importance of actin remodeling dynamics during *C. trachomatis* invasion. An additional effector, translocated membrane effector A (TmeA), was characterized to bind N-WASP at its GTPase-binding domain (GBD), which, in essence, replaces the host small GTPase Cdc42 as an activator [38,39] (Figure 1). N-WASP exists in an autoinhibited conformation, and binding of Cdc42, phosphoinositol lipids, and phosphorylation individually relieves N-WASP from this autoinhibited state to enable interaction with monomeric G-actin and the Arp2/3 complex. Unlike WAVE2 inhibition, which involves binding of several proteins to prevent interaction with the Arp2/3 complex and G-actin, N-WASP activation involves a change in conformation. While it has not been addressed specifically, TmeA binding to N-WASP GBD is thought to relieve N-WASP from its autoinhibitory conformation based on enhanced actin recruitment at the sites of invasion. This proposed mechanism is supported by *in vitro* pyrene actin assays that revealed enhanced Arp2/3-mediated actin polymerization in the presence of purified wildtype N-WASP and TmeA but not with the N-WASP ΔGBD mutant version [38]. The involvement of another effector that activates Arp2/3 via interaction with another nucleation promoting factor raises the question of why. Detailed investigation into the roles of TarP and TmeA on local actin dynamics (i.e., recruitment and turnover) revealed relatively minor contribution from TmeA [38]. Paradoxically, its effect on invasion was equivalent to that of TarP and correlated with observations of similar recruitment dynamics of the Arp2 subunit as proxy for the recruitment of the entire complex, in the Δ*tarP-* and Δ*tmeA-*mutant strains. A possibility is that the TarP- and TmeA-activated pools of Arp2/3 combine to mediate optimal actin dynamics. Another possible interpretation is the different but complementary functions of the TarP- and TmeA-associated actin networks. Monitoring the spatial distributions of recruited actin in wildtype and mutant strains lacking either TarP or TmeA demonstrated TarP to spatially restrict the formation of its actin network [37]. In the absence of TarP, TmeA-associated actin recruitment resembled mini-ruffles and blooms, which contrasted with bright actin puncta associated with wildtype and ΔTmeA-mutant strain. Therefore, despite facilitating actin recruitment, TarP and TmeA have non-redundant functions in *C. trachomatis* invasion.

#### TarP, TmeA, and dynamin-2

The non-redundance of TarP and TmeA is further highlighted with respect to the role of dynamin-2 (Dyn2) in *C. trachomatis* invasion [40] (Figure 1). Using a more sensitive quantitative imaging assays that determines the duration of internalization, which is indicated by the loss of fluorescence signal from labeled EBs at the cell surface, Dyn2 was implicated in bacterial uptake. In the same study, it was found that Dyn2 recruitment was TarP-dependent. The mechanism has not been elucidated. However, it is noteworthy that Dyn2 recruitment was not sufficient to mediate internalization. In other endocytic events, dynamins, including Dyn2, are activated to oligomerize to form collars around the necks of invaginating vesicles. The collar constricts, leading to vesicle scission from the plasma membrane [41]. In the absence of TmeA, the duration of internalization remained protracted. Surprisingly, this deficiency was corrected by the addition of a small molecular compound Ryngo 1–23, which is a known activator of dynamin oligomerization [42]. Treatment restored internalization duration to wildtype levels and importantly identified a post-Dyn2 recruitment role for TmeA. How TmeA might mediate Dyn2 oligomerization remains unknown. N-WASP is recruited along with dynamin and the Bin/Amphiphysin/Rvs (BAR) domain protein SNX9 at invaginating vesicles [43], and in this regard, TmeA/N-WASP interaction could be important. An alternative is a role for the TmeA actin network in driving membrane curvature of invaginating EB-containing vesicle, thus forming a membrane environment favorable to Dyn2 collar formation. Actin polymerization at sites of endocytosis could participate in membrane constriction at the neck of invaginating vesicles [44]. Clearly, additional studies are needed to establish the mechanism of how TarP and TmeA sequentially modulate Dyn2 function.

Prior to the identification and characterization of TmeA function, N-WASP was previously implicated in a macropinocytosis-like entry mechanism that also was dependent on SNX9 [45]. Integrating findings on actin spatial dynamics of the ΔtarP (i.e., TmeA-only) mutant, the importance of TmeA in the Dyn2 function, the involvement of the SNX9 as reported by Ford et al., and the importance of this BAR domain protein in vesicle closure and scission raise the intriguing possibility of a closure and scission of EB-containing vesicles to be pathogen-driven, and thus a part of the optimization strategy of invasion by *C. trachomatis*. However, the manipulation of Dyn2 function by TarP and TmeA is not necessary for entry, and an interesting alternative mechanisms of vesicle closure and scission could be driven by the robust actin polymerization at sites of invasion. The role of actin polymerization in this process is well documented [46].

**Perspectives**

- Invasion by the obligate intracellular *Chlamydia trachomatis* is paramount to its growth and replication and, thus, underpins pathogenesis. It enables the obligate intracellular pathogen to establish a protective intracellular niche, from where it manipulates host cell processes for its benefit.
- Central to invasion are two effectors, translocated actin-recruiting protein (TarP) and translocated membrane effector A (TmeA), which are translocated by adhered elementary bodies (EBs) to the host cell cytosol to alter signaling to the actin cytoskeleton and promote bacterial engulfment. The net result is the activation of the actin remodeling pathway of the infected cell to establish a spatially restricted actin cytoskeletal network directly underneath the invading chlamydial EB. Both TarP and TmeA sequentially regulate host Dyn2 for constriction and scission of EB-containing invaginating vesicle.
- While significant progress has been made within the past few years, there remains several unanswered questions that are fundamental to *Chlamydia* invasion, including the mechanism of selection of which invasion signaling is utilized by an EB; if and how invasion signaling is integrated with the immediate and long-term intracellular fates of nascent *Chlamydia*-containing vesicle; and how TarP- and TmeA-mediated invasions influence *C. trachomatis* interaction with macrophages and other phagocytic innate immune cells. Answers to these questions are likely to have downstream implications for *Chlamydia*–host interactions and pathogenesis, and will undoubtedly strengthen the notion that invasion underpins *Chlamydia* pathogenesis.

## Abbreviations

ABD: actin-binding domain
BAR: Bin/Amphiphysin/Rvs
CHO: Chinese Hamster Ovary
EB: elementary body
FABD: F-actin-binding domain
GBD: GTPase-binding domain
GEF: guanine nucleotide exchange factor
GTPase: guanosine triphosphatase
LD: Leu/Asp
LGV: lymphogranuloma venereum
ORF: open-reading frame
PD: phosphodomain
SH2: Src-homology 2
SNX9: sorting nexin 9
SYF: Src/Yes/Fyn
T3SS: type III secretion system
VBD: vinculin-binding domain
siRNA: small interfering RNA
